# Do solar system experiments constrain scalar–tensor gravity?

**DOI:** 10.1140/epjc/s10052-020-7721-4

**Published:** 2020-02-15

**Authors:** Valerio Faraoni, Jeremy Côté, Andrea Giusti

**Affiliations:** 1 Department of Physics and Astronomy, Bishop’s University, 2600 College Street, Sherbrooke, QC J1M 1Z7 Canada; 2 Perimeter Institute for Theoretical Physics, 31 Caroline Street North, Waterloo, ON N2L 2Y5 Canada

## Abstract

It is now established that, contrary to common belief, (electro-)vacuum Brans–Dicke gravity does not reduce to general relativity (GR) for large values of the Brans–Dicke coupling $\omega$. Since the essence of experimental tests of scalar–tensor gravity consists of providing lower bounds on $\omega$, in light of the misguided assumption of the equivalence between the limit $\omega \rightarrow \infty$ and the GR limit of Brans–Dicke gravity, the parametrized post-Newtonian (PPN) formalism on which these tests are based could be in jeopardy. We show that, in the linearized approximation used by the PPN formalism, the anomaly in the limit to general relativity disappears. However, it survives to second (and higher) order and in strong gravity. In other words, while the weak gravity regime cannot tell apart GR and $\omega \rightarrow \infty$ Brans–Dicke gravity, when higher order terms in the PPN analysis of Brans–Dicke gravity are included, the latter never reduces to the one of GR in this limit. This fact is relevant for experiments aiming to test second order light deflection and Shapiro time delay.

## Introduction

Deviations from Einstein’s theory of gravity, general relativity (GR), appear in virtually all attempts to introduce quantum corrections to gravity [1–8] (for recent overviews of GR and the challenges it faces, see, e.g., [9–11]). In addition to these deviations (in the form of extra fields, higher order terms in the field equations, and non-minimal couplings to the curvature), compelling motivation to investigate alternatives to GR comes from the 1998 discovery that the current expansion of the universe is accelerated. Within the standard $\varLambda$-Cold Dark Matter ($\varLambda$CDM) model of cosmology based on GR, one needs to introduce a completely ad hoc dark energy with a very exotic equation of state to explain the cosmic acceleration [12]. A popular alternative to dark energy consists of modifying gravity at large scales. Many modifications of GR have been proposed, the most studied being *f*(*R*) gravity [13, 14]. This is a class of theories in which the Einstein–Hilbert Lagrangian density *R* (the Ricci scalar of spacetime) is promoted to a non-linear function *f*(*R*). It turns out [15–17] that this class of theories reduces to a Brans–Dicke theory with Brans–Dicke scalar $\phi =f'(R)$, vanishing Brans–Dicke coupling parameter $\omega$, and the complicated potential $V(\phi )=Rf'(R)-f(R)\big |_{R=R(\phi )}$ (see Refs. [15–17] for reviews and [18] for extensions of *f*(*R*) gravity).

Brans–Dicke theory, originally introduced in Refs. [19–21] to account for Mach’s principle, has been generalized to the wider class of scalar–tensor theories [22–24] described by the action (we follow the notation of Ref. [25] and use units in which Newton’s constant *G* and the speed of light *c* are unity)

1 $$\begin{aligned} S_\mathrm{ST}= & {} \frac{1}{16\pi } \int d^4 x \sqrt{-g} \left[ \phi R -\frac{\omega (\phi )}{\phi } \, \nabla ^c\phi \nabla _c\phi -V(\phi ) \right] \nonumber \\&+S^\mathrm{(m)} \,, \end{aligned}$$

where the Brans–Dicke scalar $\phi$ corresponds approximately to the inverse of the gravitational coupling strength $G_\mathrm{eff}$, $\omega$ is the Brans–Dicke coupling, and $V(\phi )$ is a potential for $\phi$, which gives a range to this field. $S^\mathrm{(m)}$ is the matter action. Besides containing the cosmologically motivated class of *f*(*R*) theories, scalar–tensor gravity, which adds only a (massive) scalar degree of freedom $\phi \simeq G_\mathrm{eff}^{-1}$ to the massless spin two graviton of GR, constitutes a minimal modification of GR and is the prototype of the alternative theory of gravity [26–28]. The field equations are [21–24]

2 $$\begin{aligned} R_{ab} - \frac{1}{2}\, g_{ab} R= & {} \frac{8\pi }{\phi } \, T_{ab}^\mathrm{(m)} + \frac{\omega }{\phi ^2} \left( \nabla _a \phi \nabla _b \phi -\frac{1}{2} \, g_{ab} \nabla _c \phi \nabla ^c \phi \right) \nonumber \\&+\frac{1}{\phi } \left( \nabla _a \nabla _b \phi - g_{ab} \Box \phi \right) -\frac{V}{2\phi }\, g_{ab} \,, \end{aligned}$$

3 $$\begin{aligned} \Box \phi= & {} \frac{1}{2\omega +3} \left( \frac{8\pi T^\mathrm{(m)} }{\phi } + \phi \, \frac{d V}{d\phi } \right. \nonumber \\&\left. -2V -\frac{d\omega }{d\phi } \nabla ^c \phi \nabla _c \phi \right) \,, \end{aligned}$$

where $R_{ab}$ is the Ricci tensor and $\nabla _a$ is the covariant derivative of the spacetime metric $g_{ab}$, while $T^\mathrm{(m)} \equiv g^{cd}T_{cd}^\mathrm{(m)}$ is the trace of the matter energy-momentum tensor $T_{ab}^\mathrm{(m)}=-\frac{2}{\sqrt{-g}} \, \frac{\delta S^\mathrm{(m)}}{\delta g^{ab}}$.

Scalar–tensor gravity in the Jordan frame $\left( g_{ab}, \phi \right)$, can be reformulated in the Einstein conformal frame $( \tilde{g}_{ab}, \tilde{\phi } )$ as follows [21]. Perform the conformal transformation of the metric tensor

4 $$\begin{aligned} g_{ab} \rightarrow \tilde{g}_{ab} \equiv \phi \, g_{ab} \,, \end{aligned}$$

and the scalar field redefinition

5 $$\begin{aligned} d\tilde{\phi } = \sqrt{ \frac{|2\omega +3|}{16\pi }} \, \frac{d\phi }{\phi } . \end{aligned}$$

Since we restrict ourselves to Brans–Dicke theory with constant $\omega$, we have the non-linear scalar field redefinition

6 $$\begin{aligned} \phi \rightarrow \tilde{\phi }=\sqrt{\frac{|2\omega +3|}{16 \pi }} \, \ln \left( \frac{\phi }{\phi _0}\right) \,, \end{aligned}$$

where $\phi _0$ is an integration constant and $\omega \ne -3/2$. Both $\phi$ and $g_{ab}$ depend on the parameter $\omega$, therefore the Einstein frame metric $\tilde{g}_{ab}$ in general depends on the parameter $\omega$ (the same is true, in general, for the Einstein frame scalar $\tilde{\phi }$).

Using the Einstein frame variables $( \tilde{g}_{ab}, \tilde{\phi })$, the Brans–Dicke action (1) (with $\omega =$ const.) is rewritten as

7 $$\begin{aligned} S_\mathrm{BD} = \int d^4x\sqrt{-\tilde{g}}\left[ \frac{\tilde{R }}{16 \pi }-\frac{1}{2} \, \tilde{g}^{ab}\nabla _a\tilde{\phi } \nabla _b\tilde{\phi } -U(\tilde{\phi }) + \frac{\mathcal{L}^\mathrm{(m)}}{\phi ^2(\tilde{\phi }) } \right] \,, \nonumber \\ \end{aligned}$$

where

8 $$\begin{aligned} U (\tilde{\phi }) = \left. \frac{V(\phi )}{16\pi \phi ^2}\right| _{\phi =\phi ( \tilde{\phi }) } \,, \end{aligned}$$

where we denote Einstein frame quantities with a tilde. Formally, this is the Einstein–Hilbert action of GR with a matter scalar field with canonical kinetic energy density, but this scalar $\tilde{\phi }$ now couples non-minimally to matter. In the Einstein frame, the Brans–Dicke field equations become

9 $$\begin{aligned}&\tilde{R}_{ab}-\frac{1}{2} \, \tilde{g}_{ab} \tilde{R} = 8\pi \left( \text{ e }^{- \sqrt{\frac{64\pi }{|2\omega +3|}} \, \tilde{\phi } } \, T_{ab}^\mathrm{(m)} + \tilde{\nabla }_a \tilde{\phi } \tilde{\nabla }_b \tilde{\phi } \right. \nonumber \\&\quad \left. -\frac{1}{2} \, \tilde{g}_{ab}\, \tilde{g}^{cd} \tilde{\nabla }_c \tilde{\phi } \tilde{\nabla }_d \tilde{\phi } - U(\tilde{\phi }) \, \tilde{g}_{ab} \right) \,, \end{aligned}$$

10 $$\begin{aligned}&\tilde{g}^{ab} \tilde{ \nabla }_a \tilde{ \nabla }_b \tilde{\phi } -\frac{dU}{d\tilde{\phi }} \nonumber \\&\quad +8\, \sqrt{ \frac{\pi }{|2\omega +3|}} \, \text{ e }^{ -\sqrt{\frac{64\pi }{|2\omega +3|}}\, \tilde{\phi } } \, \mathcal{L}^\mathrm{(m)} = 0 . \end{aligned}$$

From now on we restrict to vacuum Brans–Dicke theory and set $T_{ab}^\mathrm{(m)}=0$. The explicit coupling between Einstein frame scalar $\tilde{\phi }$ and matter then disappears and the Einstein frame action (7) is formally the Einstein–Hilbert action and the Einstein frame pair $( \tilde{g}_{ab}, \tilde{\phi } )$ is formally a scalar field solution of the Einstein equations even though it has been generated by the original Jordan frame spacetime $\left( g_{ab}, \phi \right)$.

## $\omega \rightarrow \infty$ vs. GR limit

In practice, Brans–Dicke theory with $\omega =$ const. is used to approximate all scalar–tensor theories in experimental tests of gravity in the weak field regime [26–28] (this situation can be different in strong gravity when scalarization is involved, but we are not concerned with this type of situation here). It is clear that Brans–Dicke gravity reduces to GR if $\phi$ becomes constant. Precisely, the GR limit of Brans–Dicke gravity is understood as the limit in which Brans–Dicke gravity coupled to matter reduces to GR sourced by the same type of matter.

The belief that $\phi$ does so in the limit $\omega \rightarrow \infty$ is standard textbook material (e.g., [29]). However, the asymptotics of $\phi$ in this limit are important. While in most cases these asymptotics are $\phi =\phi _{\infty }+\mathcal {O}( 1/\omega )$, where $\phi _{\infty }$ is a constant [29], many analytic solutions of the Brans–Dicke field equations have been discovered over the years for which $\phi =\phi _{\infty }+\mathcal {O}( 1/\sqrt{|\omega |})$, which do not go over to the corresponding GR solutions with the same form of matter [30–37].^1^ Far from being limited to a few maverick solutions, this problem has later been shown to affect the entire electrovacuum ( i.e., $T^\mathrm{(m)}=0$) *theory* [43] and a formal explanation has been given for this “anomalous” behaviour [43–45].

Deviations from GR are well constrained experimentally in the Solar System, where gravity is weak, and to some extent also outside of it [26–28, 46, 47]. Assuming the Brans–Dicke field to be long-ranged, the best limits on scalar–tensor gravity arise from the Cassini probe and are $|\omega |> 40{,}000$ [48]. In general, experiments provide a lower bound on $|\omega |$, constraining this parameter to be large (unless $\phi$ becomes so massive and short-ranged to escape this limit, as in viable *f*(*R*) models [15–17]).

The Solar System experiments probe gravity in vacuo, the situation in which the $\omega \rightarrow \infty$ limit is anomalous. Therefore, how can experiments constraining the deviations from GR in the field of the Sun and forcing $|\omega |$ to be large, apply to a theory that does not reduce to GR in this limit? Can the parametrized post-Newtonian (PPN) approximation, which constitutes the basis for analyzing these experiments [26–28, 49–51], still discriminate between GR and Brans–Dicke gravity in the large $\omega$ regime?

This question is crucially important for experimental tests of scalar–tensor gravity, but it has not been posed in the literature thus far. Here we provide an answer: the exact (strong gravity) electrovacuum theory definitely does not reduce to GR as $\omega \rightarrow \infty$. In this limit, a (canonical, minimally coupled) scalar field survives in the limit of the field equations and acts as a matter source [52, 53]. However, the PPN analysis is limited to the weak field expansion of these field equations and, in this regime, the offending terms disappear from these equations, in which the dominant terms introduced by the scalar degree of freedom $\phi$ conform, instead, to the usual PPN analysis. This simplification occurs only to first order in the deviations of the metric and Brans–Dicke scalar from the Minkowski background, and are bound to reappear to second order and, of course, in any exact (strong gravity) electrovacuum solution of the theory. This fact is of interest for future experiments testing light deflection and Shapiro time delay to second order [54–57].

Now to the technical details. It is clear that, if the Brans–Dicke scalar becomes constant, Brans–Dicke gravity reduces to GR and therefore one should recover $\phi \rightarrow$ const. as $\omega \rightarrow \infty$. The rate at which $\phi$ approaches a constant is important. The gradient $\nabla \phi$ decays as $|\omega |$ becomes larger,

11 $$\begin{aligned} \nabla _a \phi = \phi _0 \sqrt{\frac{16\pi }{|2\omega +3|}} \, \, \exp \left( \sqrt{\frac{16\pi }{|2\omega +3|}} \,\tilde{\phi } \right) \, \tilde{\nabla }_a \tilde{\phi } . \end{aligned}$$

Consider the Jordan frame field equations and, in particular, the term which appears in their right hand side

12 $$\begin{aligned} A_{ab} \equiv \frac{\omega }{\phi ^2} \left( \nabla _a \phi \nabla _b \phi -\frac{1}{2} \, g_{ab} \, \nabla ^c \phi \nabla _c \phi \right) . \end{aligned}$$

In the literature, the failure of Jordan frame Brans–Dicke theory to reproduce the expected GR limit (which corresponds to $\phi =$ const. and to the vanishing of the right hand side of the vacuum field equations) has been reognized to follow from the fact that, when the asymptotics is given by $\phi =\phi _{\infty }+\mathcal {O}( 1/\sqrt{|\omega |})$, the tensor $A_{ab}$ does not vanish in the $\omega \rightarrow \infty$ limit but remains of order unity [30–36, 43]. It is easy to see that, when Eq. (11) is true, the tensor $A_{ab}$ reads

13 $$\begin{aligned} A_{ab}= & {} \frac{\omega }{\phi ^2} \, \phi _0^2 \, \text{ e }^{ 2\sqrt{\frac{16\pi }{|2\omega +3|}} \,\tilde{\phi } } \frac{16\pi }{|2\omega +3|} \Big ( \nabla _a \tilde{\phi } \nabla _b \tilde{\phi } \nonumber \\&-\frac{1}{2} \, g_{ab} g^{cd} \, \nabla _c \tilde{\phi } \nabla _d \tilde{\phi } \Big ) \end{aligned}$$

14 $$\begin{aligned}= & {} 16 \pi \, \text{ sign } (\omega ) \left| \frac{\omega }{2\omega +3} \right| \left( \frac{\phi _0}{\phi } \right) ^2 \, \text{ e }^{ 2\sqrt{ \frac{16\pi }{ |2\omega +3|} } } \Big ( \nabla _a\tilde{\phi } \nabla _b \tilde{\phi } \nonumber \\&-\frac{1}{2} \, \frac{\tilde{g}_{ab} }{\phi } \, \phi \, \tilde{g}^{cd} \nabla _c \tilde{\phi } \nabla _d \tilde{\phi } \Big ) \end{aligned}$$

for all values of the parameter $\omega$. Now, in the limit $\omega \rightarrow \infty$ in which $\phi \rightarrow \phi _0$, one obtains

15 $$\begin{aligned} A_{ab} \rightarrow A_{ab}^{(\infty )} = 8\pi \, \text{ sign }(\omega ) \left( \tilde{\nabla }_a \tilde{\phi } \tilde{\nabla }_b \tilde{\phi } -\frac{1}{2} \, \tilde{g}_{ab} \, \tilde{g}^{cd} \, \tilde{\nabla }_c \tilde{\phi } \tilde{\nabla }_d \tilde{\phi } \right) .\nonumber \\ \end{aligned}$$

The Einstein frame metric $\tilde{g}_{ab}^{(\infty )}$ solves the Einstein equations with the scalar field $\tilde{\phi }$ as the only matter source. This field has canonical stress–energy tensor $A_{ab}^{(\infty )}$, which is obtained as the limit of the *Jordan frame* stress–energy tensor, as

16 $$\begin{aligned} \left. 8\pi \tilde{T}_{ab}[\tilde{\phi }] \right| _{\text{ Einstein } \text{ frame }} = \left. A_{ab}^{(\infty )} \right| _{\text{ Jordan } \text{ frame } \text{ limit }} \end{aligned}$$

For $\omega >0$, this Einstein frame scalar couples minimally to the curvature and has canonical kinetic energy density. One obtains the same metric tensor by considering two candidates for a GR limit of Brans–Dicke theory: the $\omega \rightarrow \infty$ limit of the Einstein frame metric and the $\omega \rightarrow \infty$ limit of the Jordan frame metric, which coincide apart from an irrelevant positive multiplicative constant $\phi _{\infty }$. However, this metric $\tilde{g}_{ab}^{(\infty )}=g_{ab}^{(\infty )}$ obtained with these two different methods is not a solution of the vacuum Einstein equations (this would require instead $A_{ab}^{(\infty )}$ to vanish identically). Instead, $\tilde{g}_{ab}^{(\infty )}=g_{ab}^{(\infty )}$ solves the coupled Einstein–Klein–Gordon equations and, therefore, vacuum Brans–Dicke theory does not reproduce vacuum GR in the limit, as it should be for a correct “limit to GR”.

## PPN analysis and Brans–Dicke anomaly

It is well known that the only stationary, spherically symmetric, asymptotically flat black hole solution of Brans–Dicke gravity with $V(\phi ) = 0$ is the Schwarzschild metric [58–61]. If one assumes the absence of an event horizon, however, the most general static, spherically symmetric, asymptotically flat solution of the vacuum Brans–Dicke field equations with vanishing potential is parametrized by three continuous real parameters $(\alpha _0 , \beta _0 , \gamma )$ (see, e.g., [62, 63] and references therein). In detail, for $\gamma \ne 0$ the general solution reads

17 $$\begin{aligned} d s ^2 _{\gamma \ne 0}= & {} - \text{ e }^{(\alpha _0 + \beta _0)/r} \, d t^2 + \text{ e }^{(\beta _0 - \alpha _0 )/r} \, \left( \frac{\gamma /r}{\sinh (\gamma /r)} \right) ^4 \, d r^2 \nonumber \\&+ \text{ e }^{(\beta _0 - \alpha _0 )/r} \, \left( \frac{\gamma /r}{\sinh (\gamma /r)} \right) ^2 \, r^2 d \Omega _{(2)}^2 \, , \end{aligned}$$

18 $$\begin{aligned} \phi (r)= & {} \phi _0 \, \text{ e }^{- \beta _0 /r} \, \quad \beta _0 = \frac{\sigma }{\sqrt{| 2 \, \omega + 3|} }\,, \end{aligned}$$

where $d \Omega _{(2)}^2 \equiv d \theta ^2 + \sin ^2 \theta \, d \varphi ^2$, $\sigma$ denotes a scalar charge, and $4 \, \gamma ^2 = \alpha _0^2 + 2 \, \sigma ^2$.^2^ If instead $\gamma = 0$, the solution is given by the Brans class IV spacetime [62, 64])

19 $$\begin{aligned} d s ^2_{(0)}= & {} - \text{ e }^{(\alpha _0 + \beta _0)/r} \, d t^2 + \text{ e } ^{(\beta _0 - \alpha _0 )/r} \, \left( d r^2 + r^2 d \Omega _{(2)}^2 \right) , \nonumber \\\end{aligned}$$

20 $$\begin{aligned} \phi (r)= & {} \phi _0 \, \text{ e }^{- \beta _0 /r} \, . \end{aligned}$$

It is easy to see that, for this class of solutions, the scalar field approaches a constant value $\phi _\infty = \phi _0$ in the limit $\omega \rightarrow \infty$ as

21 $$\begin{aligned} \phi (r) \sim \phi _0 - \frac{\phi _0 \, \sigma }{\sqrt{2 |\omega |} \, r} + \mathcal {O}\left( \frac{1}{|\omega |}\right) \, , \end{aligned}$$

which is indeed the typical behavior for which the anomaly comes up.

Now, for $\omega \rightarrow \infty$, Eq. (17) reduces to the Fisher–Janis–Newman–Winicour–Buchdahl–Wyman metric in the limit, which is known to be the general static, spherical, asymptotically flat solution of the Einstein field equations sourced by a free scalar field $\varPhi (r) = - \sigma / (4 \sqrt{\pi } r)$, featuring a naked singularity at its centre. Alternatively, when $\gamma =0$ Eq. (19) approaches the Yilmaz geometry as $\omega \rightarrow \infty$, which is again a solution of the Einstein field equations with a scalar field $\varPhi (r) \propto 1 / r$. In other words, as $\omega \rightarrow \infty$ the family of general solutions of *vacuum* Brans–Dicke gravity discussed above *do not reduce* to a general solution of the *vacuum* Einstein field equations (namely the Minkowski space, since we have assumed the absence of event horizons). Instead, this family of solutions approaches two families of spacetimes corresponding to *non-vacuum* solutions of the Einstein field equations, thus breaking the equivalence between the GR limit of Brans–Dicke gravity and the limit $\omega \rightarrow \infty$.

It is then interesting to see how the PPN analysis of scalar–tensor theories is affected by this anomalous behavior. Using the wisdom coming from the general static, spherical, asymptotically flat non-black-hole class of solutions of *vacuum* Brans–Dicke gravity one can show that, whilst the equivalence of the two limits is not affected at the first post-Newtonian order, an effective scalar field stress–energy tensor survives at the next-to-leading order in the $\omega \rightarrow \infty$ limit. This, in turn, *prevents the full Brans–Dicke theory from reducing to GR in this limit*.

In the weak field limit the metric and scalar field are expanded as

22 $$\begin{aligned} g_{\mu \nu }= & {} \eta _{\mu \nu } + h_{\mu \nu } \,, \end{aligned}$$

23 $$\begin{aligned} g^{\mu \nu }= & {} \eta ^{\mu \nu } - h^{\mu \nu } + \frac{1}{2} \, h^{\mu \alpha } h_{\alpha }^{\,\, \nu } + \mathcal {O} (h^3) \,, \end{aligned}$$

24 $$\begin{aligned} \phi= & {} \phi _0 + \varphi + \frac{\varphi ^2}{2} + \mathcal {O} (\varphi ^3) \,, \end{aligned}$$

where $\eta _{\mu \nu }$ is the Minkowski metric and $\phi _0$ is a constant, while $h_{\mu \nu }$ and $\varphi$ are small perturbations. From Eqs. (17) and (19) one infers that

$$\begin{aligned} h_{\mu \nu } \sim \frac{\alpha _0 \pm \beta _0}{r} \end{aligned}$$

in the weak field limit. Besides, since $\beta _0 \sim \sigma / \sqrt{2 \, |\omega |}$ for large $\omega$, one can conclude that

25 $$\begin{aligned} h_{\mu \nu } \sim \frac{\alpha _0}{r} \pm \frac{\sigma }{\sqrt{2 |\omega |} \, r} \quad \text{ as } \,\,\, \omega \rightarrow \infty \, , \end{aligned}$$

which further implies that the anomaly does not show up in the weak field expansion of the left hand side of Eq. (2) since this contains only positive powers of $h_{\mu \nu }$ and its derivatives. However, the right hand side of Eq. (2) for $V (\phi ) = 0$ and in *vacuo* has a peculiar behavior. Indeed, expanding this term up to third order one finds

26 $$\begin{aligned}&\frac{\omega }{\phi ^2} \Big ( \partial _\mu \phi \partial _\nu \phi - \frac{1}{2} \, g_{\mu \nu } \, \partial _\alpha \phi \partial ^\alpha \phi \Big ) +\frac{\nabla _\mu \partial _\nu \phi }{\phi }\nonumber \\&\quad = \frac{\omega }{\phi _0 ^2} \Big ( \partial _\mu \varphi \partial _\nu \varphi - \frac{1}{2} \, \eta _{\mu \nu } \, \partial _\alpha \varphi \partial ^\alpha \varphi \Big ) \nonumber \\&\quad \quad +\frac{1}{\phi _0} \Big [ \partial _\mu \partial _\nu \varphi + \partial _\mu \partial _\nu (\varphi ^2 / 2) \nonumber \\&\quad \quad + \big (\partial _\mu h_{\nu \alpha } + \partial _\nu h_{\mu \alpha } - \partial _\alpha h_{\mu \nu } \big ) \partial ^\alpha \varphi \Big ] \nonumber \\&\quad \quad + \mathcal {O} \Big ( \varphi ^3 \, , \, h \partial \varphi ^2 \, , \, h^2 \partial \varphi \Big ) . \end{aligned}$$

Now, assuming that we work within the scenario that leads to the exact solutions discussed above and using the asymptotics (21) and (25) it is easy to see that, while at the first post-Newtonian order the scalar field contribution disappears, to second order in the PPN expansion the first term in the left hand side of Eq. (26) is $\mathcal {O} (\omega ^0)$ and survives the limit $\omega \rightarrow \infty$, breaking the equivalence between these two limits.

Let us now make more quantitative predictions using the line element (17) as an example. First, in (17) one identifies the areal radius

27 $$\begin{aligned} R (r) = \text{ e }^{(\beta _0 - \alpha _0 )/2 r} \, \frac{\gamma /r}{\sinh (\gamma /r)} \, r \, . \end{aligned}$$

Expanding for large *r*, one finds

28 $$\begin{aligned} R = r + \frac{\beta _0 - \alpha _0}{2} + \frac{3 (\beta _0 - \alpha _0 )^2 - 4 \gamma ^2}{24 \, r} + \mathcal {O} \left( \frac{1}{r^2} \right) ,\nonumber \\ \end{aligned}$$

that implies

29 $$\begin{aligned} d r^2 \simeq \left( 1 + \frac{3 (\beta _0 - \alpha _0 )^2 - 4 \gamma ^2}{12 \, r^2} \right) \, d R^2 \, . \end{aligned}$$

Hence one can implicitly recast the line element (17) in terms of the areal radius as

30 $$\begin{aligned} d s ^2 _{\gamma \ne 0} = g_{tt} \, d t^2 + g_{RR} \, d R^2 + R^2 \, r^2 d \Omega _{(2)}^2 \, , \end{aligned}$$

with

31 $$\begin{aligned} g_{tt}= & {} - \text{ e }^{(\alpha _0 + \beta _0)/r} \nonumber \\= & {} - \left( 1 + \frac{\alpha _0 + \beta _0}{r} + \frac{(\alpha _0 + \beta _0)^2}{2 \, r^2} \right) + \mathcal {O} \left( \frac{1}{r^3} \right) \, , \end{aligned}$$

32 $$\begin{aligned} g_{RR}= & {} \text{ e }^{(\beta _0 - \alpha _0 )/r} \, \left( \frac{\gamma /r}{\sinh (\gamma /r)} \right) ^4 \nonumber \\&\times \left[ 1 + \frac{3 (\beta _0 - \alpha _0 )^2 - 4 \gamma ^2}{12 \, r^2} + \mathcal {O} \left( \frac{1}{r^3} \right) \right] \nonumber \\= & {} 1 + \frac{\beta _0 - \alpha _0}{r} + \frac{3 (\beta _0 - \alpha _0 )^2 - 4 \gamma ^2}{4 \, r^2} + \mathcal {O} \left( \frac{1}{r^3} \right) \nonumber \\ \end{aligned}$$

and $$r=r(R)$$.^3^

Performing the usual PPN identifications

33 $$\begin{aligned} g_{tt} = - (1 + 2 \, \varPsi (r)) \quad \text{ and } \quad g_{RR} = 1 + 2 \, \varPhi (r) \, , \end{aligned}$$

the post-Newtonian parameter $\gamma _\mathrm{PPN}$ (not to be confused with $\gamma$) reads

34 $$\begin{aligned} \gamma _\mathrm{PPN}= & {} - \frac{\varPsi (r)}{\varPhi (r)} \nonumber \\= & {} \frac{\alpha _0 + \beta _0}{\alpha _0 - \beta _0} \left( 1 + \frac{5\alpha _0 ^2 - 6 \alpha _0 \beta _0 + \beta _0 ^2 - 4 \gamma ^2}{4 \, (\alpha _0 - \beta _0) \, r} \right) \nonumber \\&+ \mathcal {O} \left( \frac{1}{r^2} \right) \, . \end{aligned}$$

Taking the limit $\omega \rightarrow \infty$ (i.e. $\beta _0 \rightarrow 0$), one finds

35 $$\begin{aligned} \lim _{\omega \rightarrow \infty } (\gamma _\mathrm{PPN} - 1) = \frac{5\alpha _0 ^2 - 4 \gamma ^2}{4 \, \alpha _0 \, r} + \mathcal {O} \left( \frac{1}{r^2} \right) \, , \end{aligned}$$

which is always non-vanishing when the scalar charge $\sigma \ne 0$.

## Conclusions

As a conclusion, the PPN analysis narrowly escapes the problem of the GR limit arising in the full theory. It is clear, however, that this problem will reappear as soon as second and higher order terms are included in the weak field expansion and, of course, in the full strong gravity regime. To second order, the PPN analysis of scalar–tensor gravity is in jeopardy. The divergence between PPN predictions and the $\omega \rightarrow \infty$ limit of Brans–Dicke theory will then be relevant. In particular, this divergence will become important in the experimental determination of light deflection by the gravitational field of the Sun to second order in the PPN expansion [54–57]. These deviations could be obtained, in principle, with high precision astrometry, in testing strong gravity effects with the Event Horizon Telescope [65, 66] and, potentially, in tests based on gravitational waves [67–72]. Such strong gravity effects, which look more promising for detecting scalar-tensor gravity effects or further constraining the theory, will be explored in future work.

## Data Availability

This manuscript has no associated data or the data will not be deposited. [Authors’ comment: This work is purely theoretical, justifying the absence of data associated to this study.]

## Footnotes

1 Similar anomalies are occasionally reported for instances of Brans–Dicke solutions with non-conformal matter [38–42].

2 The last condition only holds for $\gamma > 0$ and there is no loss of generality in choosing $\gamma >0$ when $\gamma \ne 0$.

3 Note that for large values of *r* one has $R \simeq r$.
